# Genetic findings in miscarriages and their relation to the number of previous miscarriages

**DOI:** 10.1007/s00404-020-05859-x

**Published:** 2020-11-19

**Authors:** R. Gomez, N. Hafezi, M. Amrani, S. Schweiger, M. K. Dewenter, P. Thomas, C. Lieb, A. Hasenburg, C. Skala

**Affiliations:** 1 Klinik und Poliklinik für Geburtshilfe und Frauengesundheit, Universitätsmedizin der Johannes Gutenberg Universität Mainz, Mainz, Germany; 2 Institut für Humangenetik, Universitätsmedizin der Johannes Gutenberg Universität Mainz, Mainz, Germany; 3 IMBEI Institut für medizinische Epidemiologie, Johannes Gutenberg Universität Mainz, Mainz, Germany; 4 Vivaneo Kinderwunschzentrum Wiesbaden, Wiesbaden, Germany; 5 Kinderwunschzentrum der Universitätsmedizin der Johannes Gutenberg Universität Mainz, Langenbeckstr. 1, 55131 Mainz, Germany

**Keywords:** Early pregnancy loss, Number of previous miscarriages, Chromosomal disorders

## Abstract

**Purpose:**

Early pregnancy loss leads to a devastating situation for many couples. Genetic disorders found in the pregnancy tissue are a frequent cause of miscarriages. It is unclear whether maternal age or previous miscarriages are associated with a higher chromosomal anomaly rate. This study aimed to determine the cytogenetical distribution of chromosomal disorders in couples after one or more previous miscarriages as well as the influence of maternal age.

**Methods:**

406 fetal tissue samples obtained after spontaneous abortion between 2010 and 2014 were successfully karyotyped. This included 132 couples with at least two losses and 274 couples with sporadic miscarriage. Normal and abnormal karyotype rate was determined for age, parity, gravidity, gestational week and number of previous miscarriages by logistic regression analysis.

**Results:**

145 (35.71%) fetal tissue samples had a normal karyotype, and 261 (64.8%) did not.

After adjusting for age, older patients have a statistically significantly higher probability of genetic disorders in the pregnancy tissue (*p* < 0.001, OR 1.064, 95% CI 1.03–1.11). With each additional year, the probability of finding chromosomal abnormalities in a miscarriage increased by 6.4%. Patients younger than 35 years have a lower probability of having chromosomal disorders in the aborted material after two or more miscarriages than after sporadic miscarriages (50.7 vs. 58.9%) (*p* = 0.014, OR 0.67, 95% CI 0.48–0.914). Nevertheless, the risk of embryonic chromosomal disorders in patients aged 35 and above increased from 75.5% in sporadic miscarriages to 82.4% after more than one pregnancy losses (*p* = 0.59, OR 1.14, 95% CI  − 0.72 to 1.92).

**Conclusion:**

Chromosomal disorders found after one or more previous miscarriages are related to patients’ age. Couples suffering two or more miscarriages should be further researched, especially in younger patients.

## Introduction

Around 13% of pregnancies intended to be carried to term end with fetal loss [1]. Sporadic miscarriages are described in the literature with an incidence as between 0.88 and 5% [2, 3]. For previous miscarriages, conflicting definitions have been proposed. American and European guidelines regard the loss of two or more pregnancies as recurrent pregnancy loss (RPL) (ESHRE GDG early pregnancy loss 2017, ASRM 2013), while the WHO criteria consider three or more losses as RPL. The overall incidence under the WHO definition is around 1–3% [4–6]. Even though its prevalence seems to be lower than sporadic miscarriage, for the individuals concerned, it is an even more devastating diagnosis.

One of the most important causes of pregnancy loss found in around half of the first trimester miscarriages is fetal chromosomal abnormalities [7–10]. In case of previous miscarriages, the role of fetal chromosomal disorders needs to be better elucidated. At least half of the cases of patients suffering recurrent miscarriages are due to unknown reasons [11–13]. This fact disappoints help-seeking couples and clinicians seeking for further reasons. This emotional distress can affect future attempts to conceive because no standardized therapy can be stablished.

With the exceptions of studies by Sugiura et al. [14], Van der Berg [8], and Popescu [15], most studies about recurrent miscarriages are > 10 years old, making it difficult to compare their results with current early pregnancy detection methods. Today, the overall fetal loss and recurrent miscarriage prevalence including clinical and biochemical pregnancies might be higher.

In this study, we aimed to clarify the influence of chromosomal abnormalities on miscarriages. Therefore, we analyzed fetal chromosomal anomalies throughout the fertility lifespan found in both sporadic and after previous miscarriages.

In this retrospective approach, chromosomal analyses from 406 aborted specimens were examined. We were especially interested in evaluating the impact of chromosomal disorders as well as maternal age on sporadic and after previous miscarriages.

## Materials and methods

This is a retrospective, single-center cohort study performed by the gynecological staff at the University Hospital in Mainz.

### Population

From January 2010 to December 2014, 752 generally health patients were admitted for curettage because of miscarriage in early pregnancy. Each patient was offered a chromosomal exam with determination of the embryonic karyotype. The inclusion criteria included a sonographic presence of a gestational sac and the patient's consent to perform a chromosomal exam. In 346 cases, no chromosomal exam was performed for several reasons: no tissue growth, tissue contamination, or refusal to agree to a chromosomal exam. Patients with known genetic anomalies in either parent were not included in the study.

In total, 406 fetal tissue specimens from spontaneous abortions were obtained and analyzed. Documented parameters included parity, gravidity, maternal age, number of previous miscarriages, gestational age and cytogenetic results.

### Tissue culture and cytogenetic analysis

Upon receipt in the laboratory, chorionic villi (approximately 30 mg) were retrieved and cleaned with 0.9% NaCl solution. The probes were assessed using an inverted microscope and stored in Leibowitz media (L15) until further processing. Half of the tissue was cultivated overnight in Chang media B+C (Irvine Scientific, Giessen, Germany) at 37 °C with 5% CO_2_. It was then used for direct preparation. The remaining material was minced with a scalpel. It was split into two cultivation bottles with Bio Amf-2 media (Biological Industries, Israel) and stored at 37 °C with 5% CO_2_ for 8–14 days (long-term cultivation). The cultures were processed and stained with QFQ (Quinacrine)—banding using standard procedures. Chromosomes were analyzed with the GenASIs Bandview imaging system (Applied Spectral Imaging, ADS Biotec, Glasgow).

### Statistics

Statistics Package for Social Sciences (SPSS 22.0, Chicago IL; USA) was used for the statistical analysis.

For our overall modeling approach, we chose maternal age, parity, gravidity, number of previous miscarriages and gestational age as known relevant continuous variables to describe a pregnancy, and the outcome variable normal or abnormal embryonic karyotype to describe chromosomal factors as a cause of miscarriage.

Abnormal karyotypes were divided into numerical and structural aberrations. Numerical aberrations included trisomies, double trisomies, other trisomies (such as triple, quadruple and quintuple trisomies), sex monosomies, triploidies and tetraploidies. Mosaicisms were considered if a proportion above 10% of distinct chromosomal cell lines was found. The group others included a combination of multiple numerical and structural genetical disorders. Categorical variables were used for the descriptive analysis, summarized as total numbers, and percentages. Continuous variables were examined by reporting means with standard deviations.

The association between different continuous variables, such as age and parity, and the outcome variable presence or absence of a chromosomal disorder was studied using a linear model. For crosstabs, Fisher’s Exact Tests were performed. The significance level (*α*) was set at 5%. Odds ratios with 95% confidence intervals were used to present the results of the models. It should be considered as an explorative analysis, and as such, *p*-values are given for descriptive reasons only and should be interpreted in association with the effect estimates (OR/mean difference). To analyze the interaction between the terms age and number of previous miscarriages, we performed a separate logistic regression using the number of previous miscarriages as a continuous variable. As Independent variables, maternal age, parity, gravidity, number of previous miscarriages and gestational age were used. The OR shown was applied on a yearly basis.

## Results

The study population included a total of 406 fetal tissue specimens obtained during dilation and curettage after the diagnosis of spontaneous miscarriage. The characteristics of the patients are shown in Table 1.

**Table 1:** Patient characteristics

	All patients	Group 1 (abnormal karyotype)	Group 2 (normal karyotype)	*p* value
*N*	406	261	145	
Maternal age (years)	32.5 (±6.11)	33.35 (± 6.14)	31.0 (± 5.77)	***p*<0.001**
Parity	0.8 (±0.97)	0.86 (± 0.95)	0.7 (± 1.0)	*p* = 0.39
Gravidity	2.43 (± 1.51)	2.41 (±1.46)	2.46 (± 1.61)	
Number of miscarriages	0.49 (± 0.85)	0.45 (± 0.81)	0.57 (± 0.93)	***p*** = **0.039**
Gestational age (weeks)	10.37 (± 2.25)	10.54 (± 2.2)	10.07 (± 2.33)	*p* = 0.06
Sporadic miscarriage	*N* = 274	179 (65.3%)	95 (34.7%)	
Two or more miscarriages	*N* = 132	82 (62.1%)	50 (37.9%)	

Out of 406 aborted products, 145 showed normal karyotypes (35.7%) and 261 (64.3%) were abnormal. The most frequent abnormalities were numeric aberrations (*n* = 226) including 160 trisomies, 27 monosomies and 39 polyploidies. 18 cases showed structural aberrations only. 9 cases included mosaicisms, either alone (3 cases), combined with structural aberrations (3 cases) or combined with trisomies (3 cases). 8 cases showed a combination of multiple genetic aberrations (see Table 2).

**Table 2:** Cytogenetic findings of the fetal tissue depending on age

	< 35 years	≥ 35 years
Group 1 (abnormal karyotype)		
Numeric aberrations	*N* = 114	*N* = 112
Trisomy 13	3(30%)	7(70%)
Trisomy 16	16(41%)	23(59%)
Trisomy 18	4(66.7%)	2(33.3%)
Trisomy 21	3(25%)	9(75%)
Trisomy 22	5 (25%)	15 (75%)
Other Trisomies	28(43%)	37(57%)
Double trisomies	3(37.5%)	5(62.5%)
Sex monosomies	19(70.4%)	8(29.6%)
Triploidy	22(81.5%)	5(18.5%)
Tetraploidy	11(91.7%)	1(8.3%)
Structural abettations	*N* = 15	*N* = 3
	15 (83.3%)	3 (16.7%)
Mosaicisms	*N* = 8	*N* = 1
	8(88.9%)	1 (11.1%)
Others	*N* = 1	*N* = 7
	1 (12.5%)	7 (87.5%)
Group 2 (normal karyotype)	*N* = 106	*N* = 39
Female	52(71.2%)	21 (28.8%)
Male	54(75%)	18(25%)

Patients were divided in two groups. Group 1 comprised all patients with an abnormal embryonic karyotype in the aborted material. Group 2 comprised all patients with a normal embryonic karyotype in the aborted products.

The mean age of patients was 32.51 years (± 6.11; 17–49). Patients from Group 1 were 33.35 (± 6.14; 18–46) years old and those from Group 2 were 31.0 years (± 5.77; 17–49), respectively. Patients with a normal embryonic karyotype in the aborted products were significantly younger (*p* < 0.001).

Average gravidity in all patients was 2.43 (± 1.51) (1–10). Patients from Group 2 had 1 to 9 previous pregnancies (2.46 ± 1.61); patients from Group 1 had 1 to 10 pregnancies (2.41 ± 1.46) before. The number of miscarriages in previous pregnancies was on average 0.49 (± 0.85) (0–5).

The mean gestational age in all patients was 10.37 weeks (± 2.25) (5–21). Group 1 patients had an average gestational age of 10.54 weeks (± 2.2) (6–21), comparable to the 10.07 weeks of gestation (± 2.33) (5–20) found in Group 2 patients.

Miscarriages were considered to be sporadic if a single miscarriage was reported. 274 patients experienced a single sporadic miscarriage, whereas 132 patients experienced more than one miscarriage: 87 patients had two previous miscarriages and 45 had three or more previous losses. 243 patients were younger than 35 years and 163 patients were at least 35 years old.

### The role of previous miscarriages

The overall prevalence of an abnormal karyotype in sporadic miscarriages was 65.3% and after more than one miscarriage 62.1%. Patients with at least two miscarriages showed a lower number of chromosomal disorders than patients after one miscarriage (*p* = 0.039, OR 0.768, 95%CI 0.60–0.99 log regression, *χ*^2^). Overall, each additional miscarriage reduced the probability of chromosomal embryonic abnormalities by 23.15%.

### The role of maternal age

**Fig. 1:**
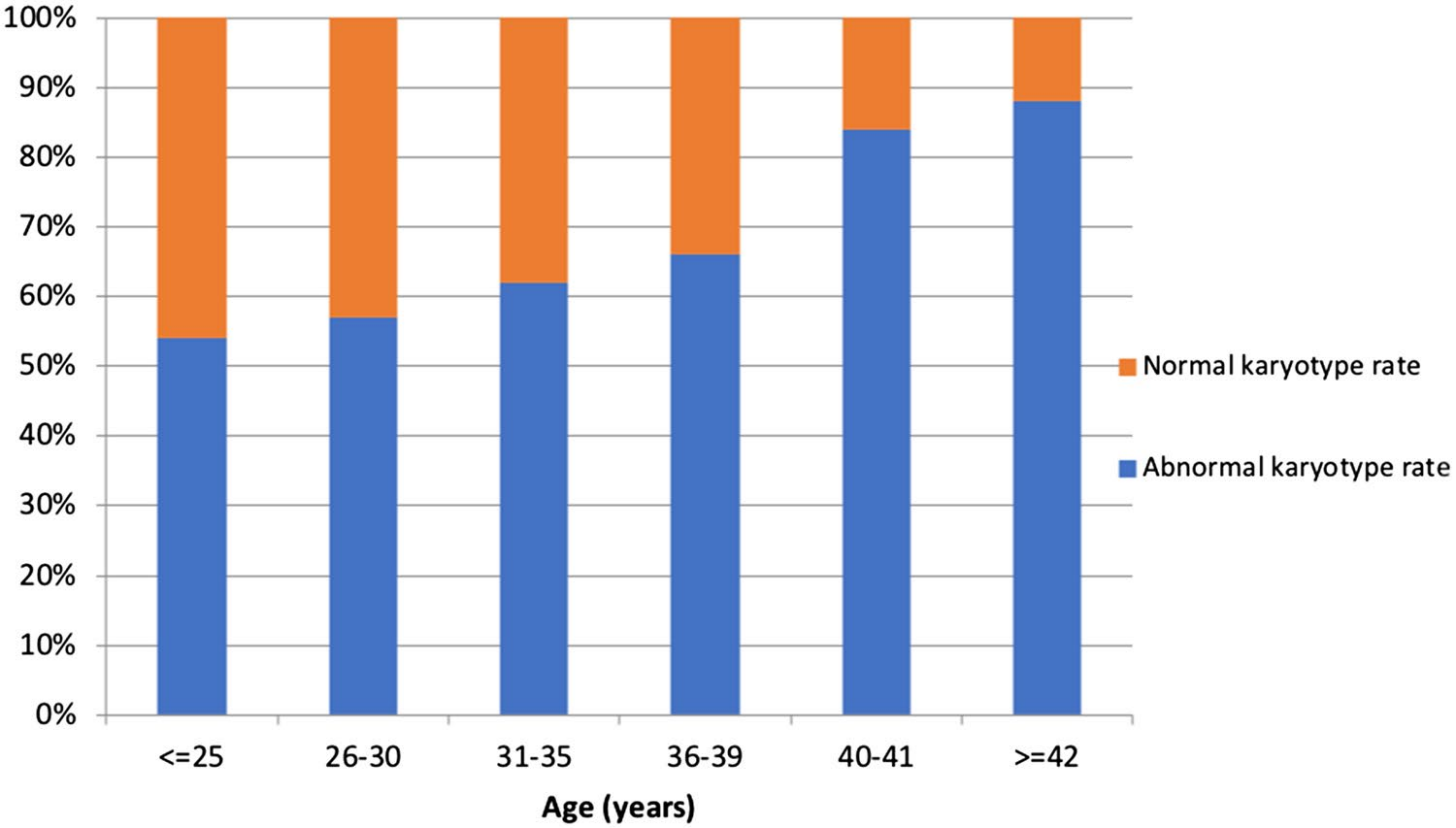
Embryonic karyotype depending on age

Maternal age was the only significant predictor of chromosomal embryonic abnormalities found (*p *< 0.001, OR 1.06, 95%CI 1.03–1.11 log regression *χ*^2^). To specify the role of age, patients were separated into two subgroups: patients younger than 35 years and patients aged 35 years and above. The embryonic anomaly rate in patients younger than 35 years was 56.4% and in patients of at least 35 years of age 76.1. In all patients, the probability of cytogenetic aberrations in the aborted material increases by 6.4% with every year. Figure 1 shows the prevalence of chromosomal abnormalities of the embryo with increasing maternal age.

## The role of maternal age and previous miscarriages

**Fig. 2:**
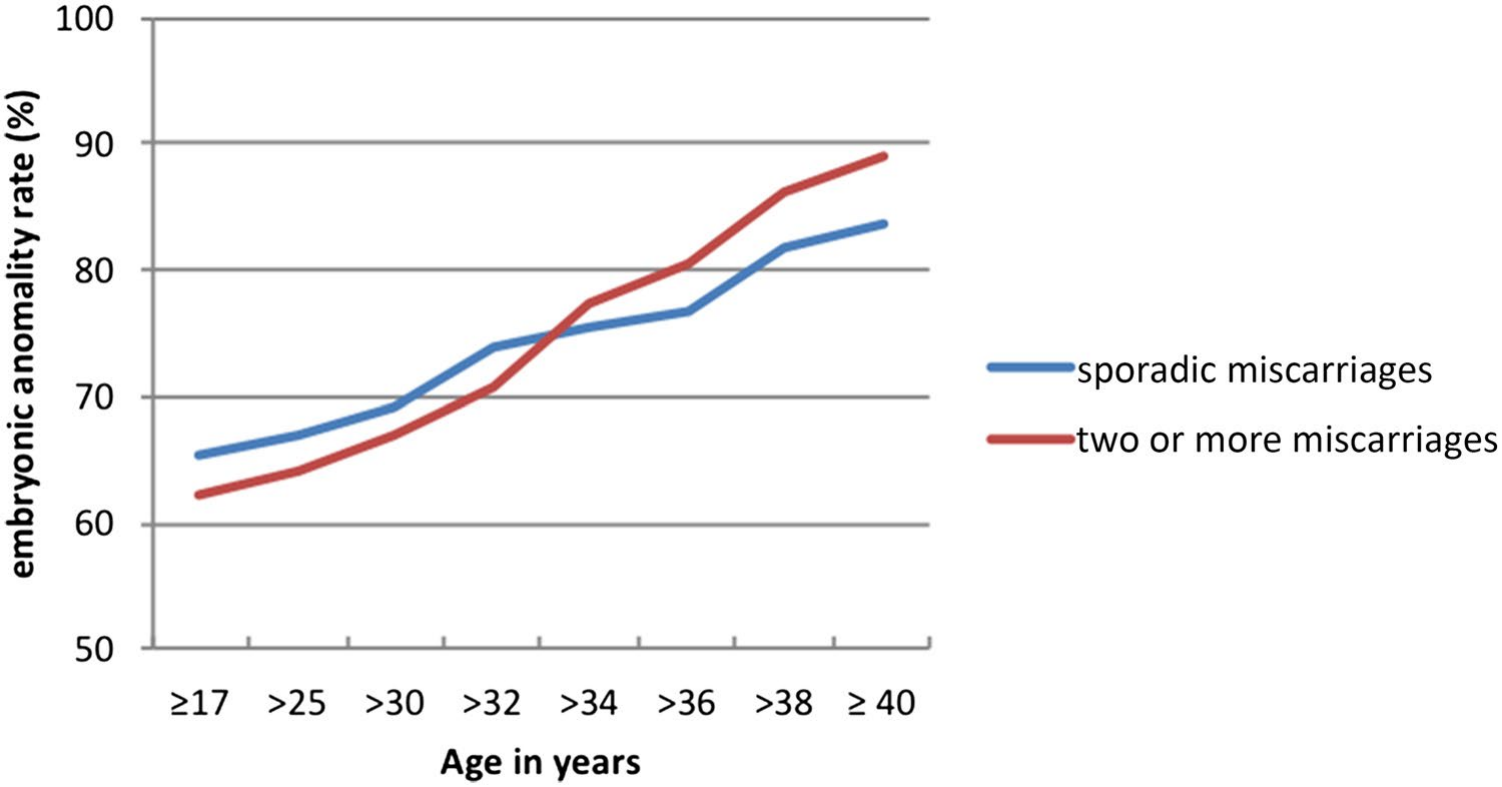
Embryonic chromosomal anomality rate depending on the age in spontaneous and after previous miscarriages

Normal/abnormal embryonic karyotypes in sporadic and previous miscarriages were estimated year by year. When focusing women younger than 35 years with and without previous miscarriages, patients with previous miscarriages showed a lower rate of abnormal embryonic karyotypes than those with sporadic miscarriages (50.7 vs. 58.9%) (*p* = 0.014, OR 0.668, 95%CI 0.48–0.91 log regression). Each further miscarriage in patients younger than 35 years reduced the probability of finding chromosomal disorder in a miscarriage by 33.16%. A lower rate of abnormal embryonic karyotypes after previous miscarriages compared to sporadic miscarriages can be seen year by year until the age of 34 (Fig. 2). In patients aged 35 years and above the number of abnormal embryonic karyotypes found in a miscarriage was higher after previous losses (82.4%) than after one miscarriage (75.5%) (*p* = 0.59, OR 1.14, 95%CI − 0.72 to 1.92 log regression).

The interaction between the terms number of previous miscarriages and age was significant (*p* = 0.03227, OR 1.061, 95%CI 1.009–1.125 log regression).

Other parameters, such as parity and week of gestation, did not influence the prevalence of abnormal embryonic karyotypes.

### Distribution of genetic disorders by age

The male/female distribution of the aborted embryos was quite similar, with 50.34% normal karyotypes in females.

No remarkable differences in chromosomal anomalies were found after one or more miscarriages among different age groups (see Table 2).

## Discussion

Pregnancy loss and especially repeated pregnancy loss are traumatic and overwhelming situations for many couples. Embryonic chromosomal disorders are a frequent and identifiable cause of early miscarriages.

This study provides an overview of genetic disorders in aborted material in a university hospital in Germany using cytogenetics.

On the one hand, maternal age has a clear effect on embryo genetics: the prevalence of abnormal embryonic karyotypes increases with advancing maternal age.

On the other hand, our study approach tried to explain the role of chromosomal abnormalities after spontaneous and after repeated miscarriages: the overall prevalence of embryonic abnormalities in sporadic miscarriages was significantly higher than after repeated miscarriages.

Analyzing maternal age, previous miscarriages and abnormal embryonic karyotypes, we could demonstrate that in women younger than 35 years, the probability to find chromosomal aberrations in embryonic tissue decreased with every miscarriage. In other words, it was less probable to find chromosomal aberrations in the embryonic tissue after two or more miscarriages than after one single miscarriage in younger patients. This inverse correlation is observed until the age of 34 years. In patients aged 35 and above the probability of an abnormal embryonic karyotype increased anyway with every miscarriage.

This study sample suggest that an abnormal embryonic karyotype is the most common cause of miscarriage [16, 17]. Compelling data suggest that the recurrence risk of chromosomal aberration mainly depends on maternal age because increasing age is associated with a higher aneuploidy rate, especially trisomy risk embryonic karyotype is the most common cause of miscarriage. The overall prevalence of chromosomal abnormalities in aborted material was 64.3% in our study. Comparably high genetic aberrations rates after miscarriages have been reported in previous publications [14, 16–19].

Maternal age can be considered as significant predictor for chromosomal aberrations in aborted material. This has been clearly demonstrated in earlier literature as well [16, 17]. Compelling data suggest that the recurrence risk of chromosomal aberration mainly depends on maternal age because increasing age is associated with a higher aneuploidy rate, especially trisomy risk. Even the absence of other risk factors, being older than 30 years is a risk factor for sporadic and repeated pregnancy loss [18–20]. In accordance with the well-known age-dependent decreasing oocyte quality associated with increasing aneuploidy rates, particularly above the age of 35 years [21], we found a higher total number of chromosomal embryonic abnormalities in the older group (at least 35 years old) compared to their younger counterparts. Our finding accords the Scandinavian study of Roepke et al. [23] with increasing prevalence of genetic aberrations found on miscarriages for the last 10 years due to the older age upon childbearing. Similar abnormality rates after three and more losses than after sporadic losses (78% vs. 70% *p* = 0.28) were reported by Marquard et al., in patients at least 35 years old [22].

The prevalence of abnormal embryonic karyotypes was higher after one miscarriage than after two and more miscarriages year by year up to a maternal age of 34 years. A real cut-off was found at the age of 35 years.

Due to the known effect of ovarian ageing in the oocyte and embryo quality, the risk of finding an abnormal embryonic karyotype increased with every miscarriage in women aged 35 years and above. Therefore, it was more probable in the older population (where the age impacts the most) that fetal chromosomal abnormalities increased year by year, regardless the number of previous miscarriages.

On the contrary, in the younger population with presumably shorter times to pregnancy and good embryo quality, every additional miscarriage might hint other pathologies related to miscarriages. Age impact here was minimized and the probability of finding chromosomal disorders was reduced by 33.16% with every miscarriage.

On the overall study sample analysis and on the younger population up to 34 years old, our finding was consistent with results from other groups reporting similar higher normal karyotypes after previous miscarriages than after sporadic losses [20, 21, 23, 24].

Interestingly enough, when focusing on our older population who showed higher chromosomal abnormalities after previous miscarriages, discordant results have been reported. Some groups found an association between the number of abortions and the chromosomal abnormalities [23, 24], while others did not [20, 22, 25, 26].

Plausible explanations for this conflicting finding may be found in the sample stratification. While some groups included patients being 35 years and above [22], other groups analyzed a wider range of ages [25, 26] acting as a confounding factor. Missing homogenous criteria for repeated spontaneous abortion (after two or after three miscarriages) as well as the imbalance in the groups´ sizes may have led to the differences observed.

There are other pathologies like thrombophilia, thyroid dysfunction, parental genetics or uterine malformations which are related to recurrent pregnancy loss and should be taken into account [27]. Since chromosomal aberrations are the most leading cause of miscarriage, the genetic analysis of aborted material is quite indispensable in case of recurrent pregnancy loss, despite the high costs.

Popescu’s group of American researchers described an overall cost-effective algorithm. Independent of age a chromosomal microarray analysis should be performed on the second miscarriage. Only after euploid results further diagnostic procedures like parental genetics, hysterosalpingography, thrombophilia, thyroid function, HbA1c and Prolactin-testing should be performed according to American guidelines. Using this algorithm, the reason for recurrent miscarriage can be found in more than 90% [9]. A similar algorithm for further diagnostics in cases of a second euploid miscarriage has been suggested by previous groups [23, 24]. The ESHRE´s European guidelines also suggest a conditional recommendation to perform genetic analysis on the pregnancy tissue only for explanatory purposes, preferring array CGH (ESHRE GDG Early Pregnancy Loss 2017).

The strength of this study is found the cohort size. This study sample had a distinct advantage in that it was composed of a defined cohort of patients with sporadic or previous miscarriages who were treated at the same institution with identical laboratory techniques, ensuring consistency of results.

Limitations to be acknowledged in this study include the missing

data about other fertility parameters or the missing cytogenetic results of preceding miscarriages or from the couples themselves. Another limitation is the well-known maternal contamination risks in female fetuses which can be found in conventional karyotyping, resulting in a higher euploid female rate [9, 28].

Specific test failure rates were unfortunately not recorded. Some reasons for culture failure might have been insufficient specimen on early weeks of pregnancy or on incomplete abortion. The high number of samples excluded for further analysis may also suggest a high culture failure when performing karyotyping. There is evidence that chromosome microarray analysis may be more effective detecting aneuploidies [29, 30]. Microarray testing has shown to be preferable over karyotyping due to a lower culture failure and it has a small incremental diagnostic yield detecting submicroscopic copy-number variations [31–33].

The findings of this study lead us to the assumption that especially patients younger than 35 years suffering from repeated miscarriages have a low probability to find chromosomal disorders in the embryonic tissue. In patients aged 35 years and above, the effect of maternal age seems to overcome the impact of the abort recurrence itself.

Chromosomal analysis should be offered after previous miscarriages before other pathologies are taken into account and further diagnostic methods are performed.

## Data Availability

The data supporting their findings can be found at the University hospital of Mainz, Langenbeckstr. 1, 55131 Mainz. All authors do consent the publication. This manuscript does not contain any individual person´s data.

## Abbreviations

ASRM: American society of reproductive medicine
ESHRE: European society of human reproduction
RPL: Recurrent pregnancy loss
